# MARS, a Multi-Agent System for Assessing Rowers' Coordination via Motion-Based Stigmergy

**DOI:** 10.3390/s130912218

**Published:** 2013-09-12

**Authors:** Marco Avvenuti, Daniel Cesarini, Mario G. C. A. Cimino

**Affiliations:** Department of Information Engineering, University of Pisa, Largo Lucio Lazzarino 1, Pisa 56122, Italy; E-Mails: m.avvenuti@iet.unipi.it (M.A.); d.cesarini@iet.unipi.it (D.C.)

**Keywords:** accelerometer, emergent approach, multi-agent systems, sports performance analysis, stigmergy, wireless motion sensing

## Abstract

A crucial aspect in rowing is having a synchronized, highly-efficient stroke. This is very difficult to obtain, due to the many interacting factors that each rower of the crew must perceive. Having a system that monitors and represents the crew coordination would be of great help to the coach during training sessions. In the literature, some methods already employ wireless sensors for capturing motion patterns that affect rowing performance. A challenging problem is to support the coach's decisions at his same level of knowledge, using a limited number of sensors and avoiding the complexity of the biomechanical analysis of human movements. In this paper, we present a multi-agent information-processing system for on-water measuring of both the overall crew asynchrony and the individual rower asynchrony towards the crew. More specifically, in the system, the first level of processing is managed by marking agents, which release marks in a sensing space, according to the rowers' motion. The accumulation of marks enables a stigmergic cooperation mechanism, generating collective marks, *i.e.*, short-term memory structures in the sensing space. At the second level of processing, information provided by marks is observed by similarity agents, which associate a similarity degree with respect to optimal marks. Finally, the third level is managed by granulation agents, which extract asynchrony indicators for different purposes. The effectiveness of the system has been experimented on real-world scenarios. The study includes the problem statement and its characterization in the literature, as well as the proposed solving approach and initial experimental setting.

## 1. Background and Motivations

The primary goal in competitive rowing is to achieve better control of velocity during the whole race. Such a goal requires highly-efficient rowing, which depends on many dynamically interacting factors. Variables, such as interpersonal coordination, seat acceleration, boat balancing and feathering should be perceived by the rower to avoid checking of the boat and wasting energy. In practice, this task is extremely difficult to perform, as it requires continuous coordination between two to eight rowers and a coxswain [1].

Conventional coaching layout in rowing consists of a coxswain in the stern and a coach in a motorboat, offering advice based on what they see and feel, based on few empirical data. With the naked eye, they can only acquire aggregate data, such as the speed of the boat, or individual data, such as the stroke rate of each rower. Suggestions are seldom precise enough to correct flaws in individual performance. For this reason, novice rowers are often taught the basics of rowing through endless hours of practice aimed at coalescing them into a team. On the other hand, professional rowing races are typically decided by the order of tenths of seconds. Hence, a computer-aided approach to improve the training process in rowing is highly desirable [2].

The classical way a rower can evaluate his individual performance is by indoor rowing machines equipped with a software system [3]. Several studies on the monitoring of rowing have reported on the factors influencing performance. As a general remark, most studies and systems concerned with the recording of rowing biomechanics provide only some basic measurement and simple statistical analysis tools to assist the coach and the athletes on the training phase [4–6].

In the last decade, wearable sensors and wireless sensor networks (WSNs) have been applied to monitor human movements [7–11] and, more specifically, to obtain high-resolution real-time parameters on rowing performance [12]. Most of the initial efforts have been concentrated on data acquisition and integration [13,14]. Recently, some experimental studies have started to address the problem of providing real-time feedback and on-water analysis of the biomechanics indexes of the athletes during training. In practice, monitoring the crew performance in real-time requires choosing a trade-off between what to monitor and how to present it [15]. Indeed, there are many possible parameters, and their tracking should be related to the specific training practice, according to a process-oriented approach. Actually, many efforts in the field have been aimed at supporting system-oriented analyses based on complex mathematical models of the rowing performance. One of the most important lessons learned from these efforts is that the algorithms used to perform the parametric aggregation must use a limited amount of states, be highly flexible and be able to handle noise. Indeed, much work still has to be done before such systems can be used on a regular basis for monitoring crew team performance [4].

A novel perspective can be gained by considering a different design paradigm. It has been argued that any explicit modeling of a collective behavior effectively biases the system and constrains it within an idealized description that is dependent on the *cognitive* requirements of the designer [16]. Typically, this approach deploys an arsenal of techniques, including machine learning and probabilistic modeling, in the attempt to deal with the inherent uncertainness, time-varying and incomplete nature of sensory data. However, this does not alter the fact that the representation of a functional structure is still predicated on the descriptions of the designers. In contrast, with an *emergent* approach, collective perception is concerned with the augmentation of sensory data in order to enable local action. It is not a process whereby the observation of an external observer is abstracted and represented in a more or less symbolic manner, *i.e.*, the so-called *cognitivist* approach [16]. Emergent paradigms are based on the principle of self-organization [17], which means that a functional structure appears and stays spontaneous at runtime. The control needed to achieve results is distributed over all participating entities. In the literature, the mechanisms used to organize these types of systems and the collective behavior that emerges from them are known as *swarm intelligence, i.e.*, a loosely structured collection of interacting entities [18].

An emergent system is intrinsically embodied, and its physical instantiation plays a direct constitutive role in its lifecycle [16]. There are two complementary aspects in the embodiment: the self-organization of the system and the coupling of the system itself with its environment. Emergent behavior is then inherently specific to the embodiment of the system and dependent on the systems history of interactions, *i.e.*, its experiences. Hence, an emergent system cannot be specified and designed as a separate part with respect to its application domain. In contrast, with cognitivist systems, there is a dualist distinction between the computational processes and the computational infrastructure and devices that effect any physical interaction. Indeed, cognitivism asserts that external reality can be modeled and embedded in the system by a human designer.

Emergent approaches represent the application of biologically-inspired patterns to software design. The purpose is to overcome designer-dependent representations of a system, which are more efficient, but work, as long as the system does not have to stray too far from the conditions under which these explicit representations were formulated. By using emergent paradigms, the collective properties or interactions between the parts of a complex system can be described in terms of the properties of individual agents that interact with the environment and whose behavior is specified by it. In contrast, cognitivism involves a view of cognition that requires the representation of a given pre-determined objective established on the basis of domain knowledge acquisition in the design process. Hence, a cognitivist system can be characterized for its efficiency in solving a specific application problem with more or less adaptability, in contradistinction with an emergent system, which is characterized by adaptation, autonomy and self-organization.

The fact that simple individual behaviors can lead to a complex emergent behavior has been known for decades. More recently, it has been noted that this type of emergent collective behavior is a desirable property in pervasive computing [18,19]. In [18], a number of application scenarios from a range of different domains have been reported. Such scenarios are supported by the stigmergic paradigm in order to build self-coordinating environments that promote the autonomy of entities and provide robust behavior. The evaluation is used to demonstrate how a model based on stigmergy can be used to provide a highly-decentralized method of organizing the components of a pervasive computing environment. In [19], the authors present an agent-based framework, in which cooperative software agents find solutions to back-end tracing problems by self-organization. Such cooperative agents are based on a business process-aware traceability model and on a service-oriented composition paradigm. Furthermore, an interface agent assists each user to carry out the front-end tracking activities. Interface agents rely on the context-awareness paradigm to gain self-configurability and self-adaptation of the user interface and, on ubiquitous computing technology, *i.e.*, mobile devices and radio-frequency identification, to perform agile and automatic lot identification. Biological paradigms have inspired significant research, not only in robotics and communication networks, but also in pattern detection and classification. For example, in [20], a number of agent-based architectures for distributed pattern detection and classification are presented. More specifically, basic components of such systems, their different strategies and the different types of agents are studied. The study demonstrates important properties, such as robustness, scalability and fast convergence.

According to the *stigmergy* paradigm [21,22], agents do not communicate with each other, but indirectly interact by changing their environment. In biology, stigmergy is a class of mechanisms that mediate animal-animal interactions. It consists of indirect communication that is taking place between individuals of an insect society by local modifications induced by these insects on their environment [23]. The term is formed from the Greek words *stigma* and *ergon*, which mean *sign* and *action*, respectively, and captures in the information processing field the notion that an agents actions leave signs in the environment, signs that it and other agents sense and that determine their subsequent actions. In the literature, various types of stigmergy have been distinguished. *Sign-based* stigmergy occurs when markers are left in the environment to influence the subsequent behavior (choice and parameters) of entities. In *quantitative* stigmergy, the mark varies in a quantitative manner. In a stigmergic computing scheme, the environment acts as a shared medium through which agents communicate. Each agent is able to sense and change the state of a part of the environment. These changes need to persist long enough to affect the subsequent behavior of other agents. Hence, the environment acts as a common shared service for all entities, enabling a robust and self-coordinating mechanism [23,24].

This paper describes and discusses how an emergent approach can be used for measuring both the overall crew asynchrony and the individual rower asynchrony towards the crew. Based on this approach, a prototype of a tool for assisting the coach in perceiving the crew's coordination on water has been implemented and demonstrated experimentally. The results of this *in situ* experiment are presented and discussed. The approach and the prototype are referred to as MARS (multi-agent system for assessing rowers' coordination via motion-based stigmergy). Agents of the system use stigmergy as the coordination mechanism.

In [24], we presented a multi-agent system for the detection of situations related to social events via position-based stigmergy and fuzzy rules. The proposed system is managed by different agents in order to recognize situations through inference of fuzzy rules. Antecedent and consequent parameters of fuzzy rules are defined by means of an adaptation procedure. The system was tested on real-world meeting scenarios involving a different number of participants. The obtained results in terms of situation detection and responsiveness show that the proposed scheme can be successfully applied to recognize situations in any scenario regardless of the number of participants.

The paper is organized as follows. Section 2 covers the related work on performance analysis in rowing. In Section 3, we introduce the architecture of the MARS system. Section 4 is devoted to the multi-agent model of processing with its related stigmergic paradigm. Section 5 describes the deployment of the MARS system architecture. In Section 6, we discuss experimental results. Section 7 draws some conclusion and suggests future work to be undertaken.

## 2. On-Water Rowing Monitoring: Related Work

To the best of our knowledge, no work has been done in the field of rowers asynchrony processing using an emergent approach and wireless sensors. However, there are a number of projects that measure raw biomechanical parameters of rowing and perform analysis using a cognitivist approach. In this section, we intend to present such projects with the aim of providing a landscape of the current methodologies. Moreover, the comparison presented in this section does not take into account hardware settings (e.g., number and types of sensors) and performance indicators.

In [2], the experience of the application of WSN for rowing performance was presented. The system was able to monitor boat speed and set and the synchrony of the rowers based on their seat acceleration. Since acceleration data contains a high noise ratio, with a cognitivist approach, only a few data points are of practical interest, namely, maximum acceleration during stroke and maximum deceleration during finish. The local computation is then mainly devoted to extract and report critical points and to calculate some aggregation, e.g., frequency of oscillation. Such extracted data are then sent to a display. To cope with the highly variable and noisy character of such data, the authors employ a dynamic calibration algorithm in the parameters' setting. However, the authors claim that such an algorithm is not sensitive enough and might be fine-tuned for use on a regular basis.

In [13], the authors have applied WSN to the oars and boat for monitoring boat movement, boat balancing and the trajectory of the stroke. The novelty of the approach consists in the usage of a couple of accelerometers placed on each oar in order to calculate its angular velocity. Three different tests are performed: an early calibration in the laboratory, an indoor experiment with an ergo-meter and on-boat trials. The resulting data is stored in a file. Subsequent analysis of such data is not performed, as the authors have performed this test and data collection only as a proof-of-concept of their technology.

In [25], the authors presented a coaching device for rowing and an analysis software, called Accrow and Regatta, respectively. Accrow employs an accelerometer and a GPS receiver to measure the boat acceleration and velocity, respectively. Regatta analysis provides boat velocity, stroke rate, propulsion per stroke, distance traveled by the boat and the required running times. The coach receives such performance data at the end of the on-water training unit. The system made of Accrow and Regatta can be used to analyze the effects of different rowing techniques or different stroke rates on the boat velocity.

Geospatial data has been used by [4] for measuring rowing performance in terms of boat velocity and acceleration variation of a single stroke cycle. In particular, the study provides a classification of physical parameters in four categories, depending on their source, and a conceptual approach for monitoring and evaluating rowing. Some preliminary tests are discussed, focusing on the potential of mobile mapping technology, on the various data types, the sensors, their level of integration and limitations. The testing is carried out with a data acquisition system made of an acceleration sensor and a GPS receiver. However, the system is focused on stroke cycle characterization in terms of acceleration and speed.

In [14], an integrated data acquisition system for rowing performance analysis was presented. The analysis is carried out by means of post-processing. The authors point out that a great deal of effort is necessary for the in-field calibration procedure, which is supported by an *ad hoc* software directly interfaced with the tool used to handle the signals. The paper describes the design, calibration and evaluation of a broad range of sensing devices placed on the boat. The study is focused on designing innovative rowing shells meeting the specific requirements of a crew.

In [26], a WSN-based approach to improve rowing performance was presented. The authors describe the design of the system and some real-world experiments. They investigate how to integrate inertial measurement units into the process of rowing technique optimization. The study is focused on possibilities offered by the sensors and employs conventional signal processing techniques, giving some insights about the type of sensors to be used. The system has been experimented upon in both training and racing conditions, showing its ability to measure rowing technique indicators, such as stroke length and stroke rate, for both amateurs and world-class rowers.

In [27], the authors presented an evaluation of online sonification as an aid for visually-impaired rowing athletes. The approach allows athletes to better follow the movements of the rest of the rowing team. The system, called Sofirow, is implemented on a device that samples accelerations and the speed of the boat and produces a parametric sound, directly proportional to the linear acceleration of the boat. Thus, the perception of the boat run by the athletes is enhanced, as the single rowing cycle can be perceived as a short sound sequence.

In [15], a quantitative evaluation of four different sonification schemes for rowers was presented. The study is considered more extensive than the work carried out in [27], as a broad range of sonification models are used, *i.e.*, wind, pure tone, musical instruments and car engine. Questions about the characteristics of the sound stimuli are also posed in order to assess the ability of the participants to extract information from the sonification models.

## 3. The Overall Architecture

In this section, we first provide an ontology-driven conceptual modeling, so as to sufficiently capture the most important requirements and tasks to be performed; then, we detail the main modules of the system.

### 3.1. An Ontological View of the Proposed Approach

**Figure 1.**
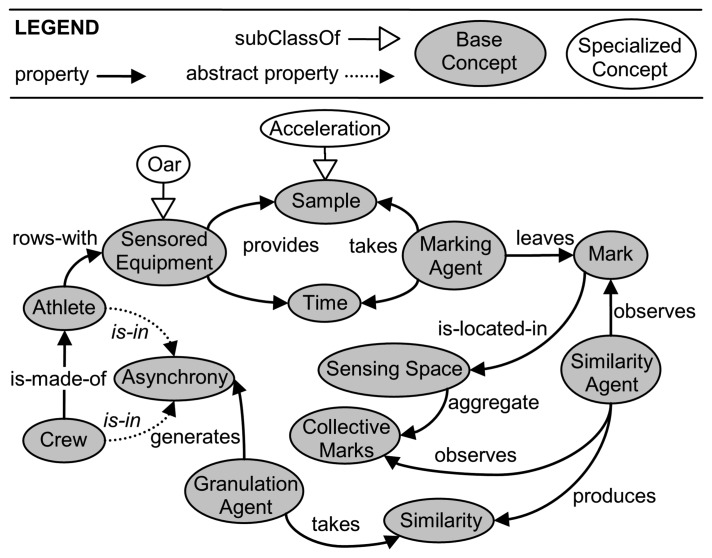
An ontological view of the emergent approach for measuring asynchrony in rowing.

An ontological view of the MARS system is represented in Figure 1, where base concepts, enclosed in gray ovals, are connected by properties, represented by black directed edges. The core properties are *Athlete is in Asynchrony* and *Crew is in Asynchrony*. As these properties cannot be directly sensed (*i.e.*, instantiated) by the system, they are *abstract* properties, shown by dotted edges. The overall system is aimed at indirectly discovering them, by observing the collective strokes of the rowers starting from data provided by wireless sensors.

More formally, let us consider a *Crew* of *N Athletes*, each of them rowing with *Sensored Equipment*. As a specific sensored equipment, we considered the *Oar* (in figure, specific properties are shown with white ovals and are connected by white directed edges). A *Sensored Equipment* constantly provides *Samples* and related *Time*, which are taken by a *Marking Agent*. As a specific sample, we considered the *Acceleration*. Each *Marking Agent* leaves *Marks*, which are located in the *Sensing Space*. For each *Sensored Equipment*, there is a *Marking Agent. Marks* are aggregated in the *Sensing Space*, generating *CollectiveMarks*. For each *Crew*, a *Similarity Agent* observes *Marks* and *Collective Marks* in order to produce a *Similarity* measure of them with respect to optimal marks; these correspond to the marks produced under a desired level of synchrony. Finally, a *Granulation Agent* takes as input the *Similarity* and generates a level of *Asynchrony*.

### 3.2. The Main Modules of the MARS System

The MARS system is made of four main subsystems: (i) a sensing subsystem, *i.e.*, wireless sensor nodes (motes) placed on oars to allow local sensing of the strokes; (ii) a tracking subsystem, which collects the sensed data; (iii) a processing subsystem, which computes the performance indexes; and (iv) a displaying subsystem, which provides the performance indexes to the coach. In terms of hardware components, the setting of the sensing subsystem employs accelerometers, whereas the other subsystems run on a conventional laptop.

**Figure 2.**
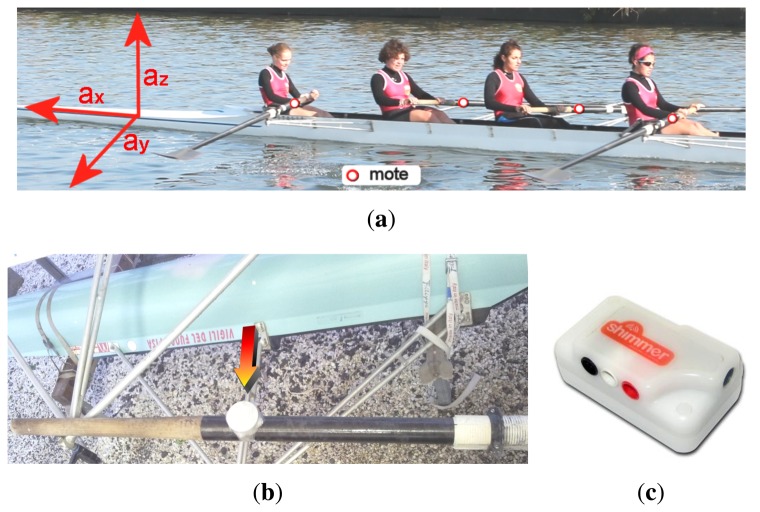
(**a**) Position of the motes on the boat; (**b**) a sensored oar; (**c**) a mote.

The sensor placement and a sensored oar are shown in Figure 2. To allow noise reduction in the data produced by sensors, motes have been placed on the inboard segment of the oar, between the handle bottom and the oarlock. To improve accuracy, simple techniques may be used to virtually align the motes with respect to a reference system [28]. In Figure 2b, the oar and a waterproof enclosure endowing the mote are shown. Figure 2c shows one mote used for the experiment, a COTS (Commercial Off-The-Shelf) device sold by Shimmer research, running the TinyOS, an embedded OS.

Figure 2a also shows a tri-axial reference system for acceleration, *a_x_*, *a_y_* and *a_z_*, parallel to the longitudinal, horizontal and vertical axes of the boat, respectively. The asynchrony of rowers can be measured based on two phases, which are both carried out along the vertical axis: (i) the placing of the oar blade in the water and (ii) the removal of the blade out of the water. For this reason, we will consider only the vertical axis, *a_z_*, referred to as *a*, for brevity. This mono-dimensional input signal will also allow us a simple and effective presentation of the method.

**Figure 3.**
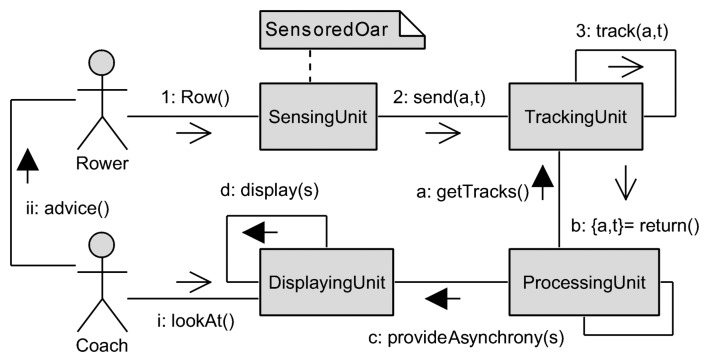
Communication diagram of the main modules of the multi-agent system for assessing rowers' coordination via motion-based stigmergy (MARS) system.

The main modules of the MARS system are represented in Figure 3, by means of a communication diagram. Here, an interaction organized around the users (shown as stick figures) and the parts of the system (shown as rectangles) is represented. In particular, synchronous and asynchronous messages are shown with filled and stick arrowheads, respectively. Finally, a comment is shown as a rectangle with a bent upper-right corner. The interaction starts with the *Rower* who interacts with the *Sensing Unit* by means of the sensored oar (1). The *Sensing Unit* periodically sends data (e.g., acceleration and timestamp) to the *Tracking Unit* (2), which is responsible for tracking all sensed data (3). The *Processing Unit* periodically takes from the *Tracking Unit* (a) a batch of sensed data (b), and provides the *Displaying Unit* with asynchrony measures (c). The *Displaying Unit* provides such asynchrony measures to the *Coach* (d). When needed, the *Coach* looks at the *Displaying Unit* (i) and advises the *Rowers* (ii), who correct their rowing activity accordingly, and so on.

In the following section, we provide a more detailed view of the peripheral units. The core of the system is represented by the *ProcessingUnit*, which is described and analyzed in Section 4.

### 3.3. The SensingUnit Module

The sensing unit is made of a Shimmer mote attached to the oar. The size of a mote is 53 × 32 × 25 mm. Each mote is composed of a micro-controller, a rechargeable battery, a three-axis accelerometer, a Bluetooth transceiver and other components not relevant to this work. The mote runs a program written in NesC (http://nescc.sourceforge.net) over TinyOS (http://www.tinyos.net), an open source, event-driven operating system designed for networked embedded sensor systems. The mote has been programmed so as to be managed with minimum power consumption. More specifically, it cyclically samples the accelerometer at a given frequency and sends the sampled data to the tracking unit. The sampling frequency can be set via wireless communication, by using a simple two-way protocol. The mote can also be reset and synchronized upon commands sent using a wireless transceiver. We experienced that, with this management program, the average battery life was three hours, thus allowing us to perform long-lasting on-field tests. A limit of such motes is the relatively short transmission range of their on-board Bluetooth transceiver, which is roughly 20 m.

### 3.4. The TrackingUnit Module

The tracking unit is made of a laptop equipped with a Bluetooth receiver. The logic of the unit is developed in Java. The unit is responsible for wireless interactions with the motes, *i.e.*, reset mote, set sampling frequency and synchronize mote. At the physical level, the unit works as a Bluetooth master to the motes. At the application level, when the application starts, the unit forces a preliminary synchronization of the motes, through a simple three-way handshake protocol. While the system is running, some clock drifts on the motes may occur. Hence, timestamps provided by motes may not be synchronized with respect to the tracking unit. To overcome this issue, timestamps are adjusted on the tracking unit, considering the sampling period and the inter-arrival time of the samples.

### 3.5. The DisplayingUnit Module

The MARS system supplies the coach with both visual and aural [29] displays. The visual channel provides quantitative values of asynchrony, whereas the aural channel is designed for providing qualitative information. The aural channel is more immediate and has the advantage of leaving the coach free from watching the asynchrony data continuously. This allows him to follow the evolution of the whole team or the performance of a single rower with respect to the team in an easier and more effective way.

With the MARS system, the coach is able to access the asynchrony via the following use cases: (i) listen to the individual asynchrony; (ii) watch the individual asynchrony; (iii) listen to the collective asynchrony; and (iv) watch the collective asynchrony. Advice to rowers can be provided by the coach in a conventional manner, *i.e.*, with his own voice. During a training session, the coach, who is normally tuned via the aural channel, looks at the visual content only occasionally. At the end of the training session, he can examine the visual plot of individual and collective asynchrony, in order to establish a performance improvement initiative for the next session.

## 4. The *ProcessingUnit* Module

In this section, we first introduce some definitions to formalize our method. Then, we describe the different processing phases.

### 4.1. The Static Pre-Filtering

**Figure 4.**
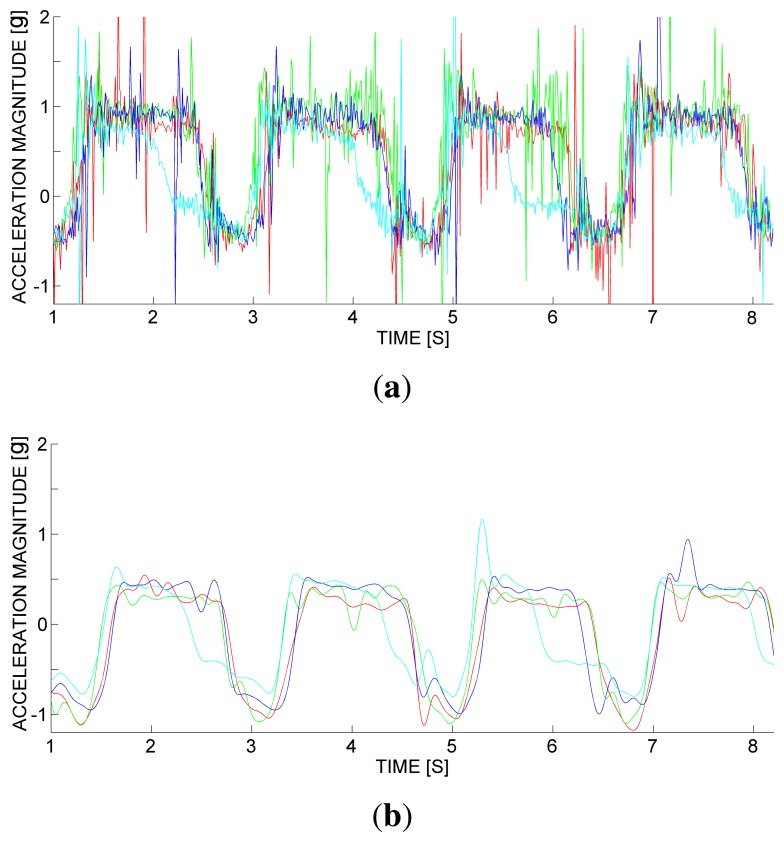
A pilot scenario of vertical acceleration magnitude against time, with 4 sensored oars: (a) raw samples; (b) after a low-pass filtering with a cutoff frequency of 20 Hz.

Let us consider an equipment of *N* wireless accelerometers, one for each rower, releasing an acceleration magnitude vector sample ${\text{a}}^{\left(i\right)}=(a_{1}^{\left(i\right)},\ldots ,a_{N}^{\left(i\right)})$ every *T_S_* seconds, where $a_{j}^{\left(i\right)}\in \mathbb{R}$, *j* = 1, …, *N* and *i* ∈ ℕ. Figure 4 depicts a pilot scenario of vertical acceleration magnitude (normalized to gravity *g*) against time, for *N* = 4 rowers (each *j*-th channel represented with a different color).

More specifically, Figure 4a depicts raw signals, containing some electro-mechanical noise caused by micro-scale effects that are independent of human activity. To remove the electro-mechanical noise, a static filtering is first performed. The static filtering is made of classic low-pass FIR (Finite Impulse Response) filters, namely, a Hamming-window based, linear-phase filter. We employed a cutoff frequency of 20 Hz. With this parameter value, the filtering causes a delay of a few tenths of a second, which is negligible for the application domain. We also made the signal zero-mean by removing the average, which is irrelevant for detecting the rowing stroke. Figure 4b shows the effect of the static filtering on the raw samples in the pilot scenario.

### 4.2. The Marking Processing Level

**Figure 5.**
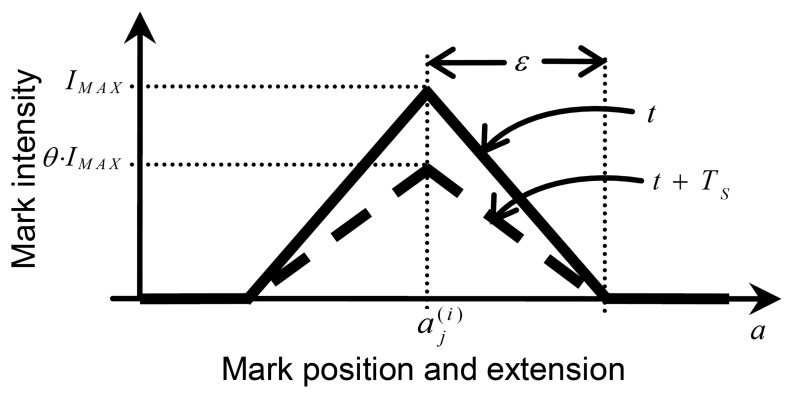
A single triangular mark released in the sensing space by a marking agent (solid line), together with the same mark after a step of decay (dashed line).

While the *j*-th athlete is rowing, for each sample, $a_{j}^{\left(i\right)}$, released at the *i*-th step, the marking agent related to the athlete's oar deposits a mark in the sensing space. Figure 5 shows a triangular mark (with solid line), which is characterized by a central (maximum) intensity, *I_MAX_*, an extension (or spatial decay), *ε*, and a temporal decay, *θ*, with *ε* > 0 and 0 < *θ* < 1. Here, *ε* and *I_MAX_* are the half base and the height of the triangular mark, respectively. Figure 5 shows, with a dashed line, the same mark after a period of decay, *T_S_*. The mark intensity spatially decreases from the maximum, corresponding with the acceleration value of $a_{j}^{\left(i\right)}$, up to zero, corresponding with the acceleration value of $a_{j}^{\left(i\right)}\pm \varepsilon$. Further, all the intensity released has a temporal decay, of a percentage, *θ*, per step, as represented with the dashed line. More precisely, *θ* corresponds to a proportion of the intensity of the previous step. Hence, after a certain decay time, the single mark in practice disappears. The decay time is longer than the period, *T_S_*, by which the marking agent leaves marks. Thus, if the athlete holds an approximately constant acceleration, at the end of each period, a new mark will superimpose on the old marks, creating an accumulated mark, whose intensity will reach a stationary level. In contrast, if the marking agent moves to other accelerations, the mark intensities will decrease with time without being reinforced.

More formally, at the *i*-th instant = *t*^(^*^i^*^)^ = *i* · *T_S_*, the *j*-th marking agent leaves in the sensing space a mark of intensity $I_{j}(a,t^{\left(i\right)})\equiv I_{j}^{\left(i\right)}(a)$ defined as:

(1) $$I_{j}^{\left(i\right)}(a)=\max\;\left(0,I_{\text{MAX}}\cdot \left[1-\varepsilon^{-1}\cdot \left|a-a_{j}^{\left(i\right)}\right|\right]\right)$$

Every *T_s_* seconds, the intensity of the mark released at the *i*-th instant automatically decays of a percentage *θ* of its current value, that is:

(2) $$I_{j}^{\left(i\right)}(a,t)=u(t-i\cdot T_{S})\cdot I_{j}^{\left(i\right)}(a)\cdot \theta^{\frac{t-i\cdot T_{S}}{T_{S}}}$$

where *u*(*t*) is the unit step function, *i.e.*, a discontinuous function, whose value is zero for a negative argument and one for a positive argument.

In order to assess whether the superimposition of marks yields the maximum intensity level to converge to a stationary level, let us consider a theoretical scenario that produces the utmost possible intensity level. In such a scenario, the *j*-th marking agent keeps its value of acceleration, ${\bar{a}}_{j}^{\left(i\right)}$ constant and releases an infinite series of identical marks, with a temporal period of *T_S_* seconds. Hence, the current intensity level, *I_j_*(*a*, *t*), of the accumulated mark is obtained as the sum of the intensities of the marks left by the *j*-th marking agent, that is, from Formula (2):

(3) $$I_{j}(a,t)=\overset{Z=\left\lfloor t/T_{S}\right\rfloor }{\underset{i=0}{\text{∑}}}u(t-i\cdot T_{S})\cdot I_{j}^{\left(i\right)}(a)\cdot \theta^{Z-i}$$

Then, from Formula (3), we can deduce that after *Z* · *T_S_* seconds:

(4) $$I_{j}(a,t)=I_{j}^{\left(0\right)}(a)\cdot \theta^{Z}+I_{j}^{\left(1\right)}(a)\cdot \theta^{Z-1}+\ldots +I_{j}^{\left(Z\right)}(a)$$

Since $a_{j}^{\left(i\right)}={\bar{a}}_{j}^{\left(i\right)}$ is constant, from Formula (1), it follows that $I_{j}^{\left(0\right)}(a)=I_{j}^{\left(1\right)}(a)=\ldots =I_{j}^{\left(Z\right)}(a)$. As a consequence, Formula (4) becomes the sum of the first *Z* + 1 terms of a geometric series, and it follows that:

(5) $$I_{j}(a,t)=I_{j}^{\left(0\right)}(a)\cdot \frac{1-\theta^{Z+1}}{1-\theta }$$

If *Z* ≫ 1, then:

(6) $$I_{j}(a,t)\to I_{j}^{\left(0\right)}(a)\cdot \frac{1}{1-\theta }$$

For instance, with *θ* = 0.75, the stationary level of the maximum is equal to 4 · *I_MAX_*.

**Figure 6.**
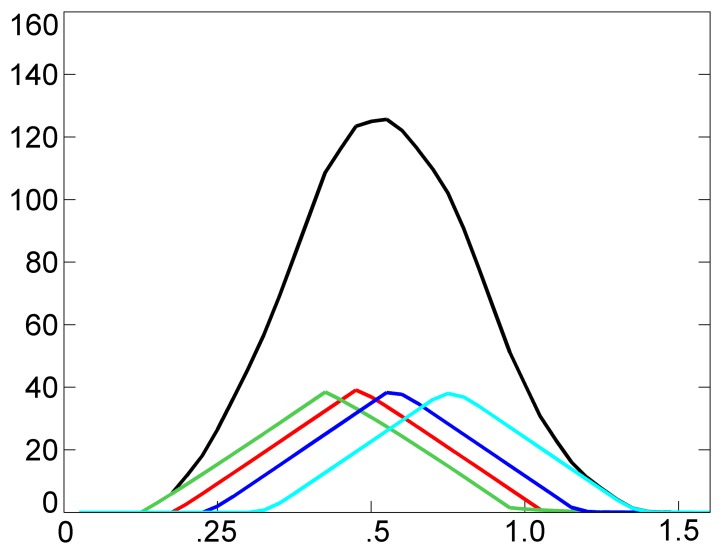
An example of four accumulated marks (colored lines) and of a collective mark (black line) created in the sensing space at the instant t = 3.22 s by the signal of Figure 4 (c) (pilot scenario), with *I_MAX_* = 10, *ε* = 0.3 and *θ* = 0.75.

Analogously, when superimposing identical marks of *N* rowers, we can easily deduce that the intensity of the collective mark grows with the passage of time, achieving a collective stationary level equal to *N* times the level of Formula (6). Figure 6 shows an example of four accumulated marks (colored lines) and of the collective mark (black line) created in the sensing space at the instant, *t* = 3.22 s, by the filtered signal of Figure 4c, with *I_MAX_* = 10, *ε* = 0.3 and *θ* = 0.75. It is worth noting that the accumulated marks have a triangular shape, with their maximum value close to *I_MAX_*/(1−*θ*) = 4·*I_MAX_*. It can be deduced that in the recent past, the acceleration was almost stationary for all rowers. As a consequence, also the collective mark has a shape close to the triangular one.

### 4.3. The Similarity Processing Level

The first important observation from the marking processing level is that an accumulated mark takes a triangular shape when acceleration does not vary sensibly within the last steps. We suppose here that any rowing stroke produces a signal whose acceleration pattern strongly depends on a number of factors. In our approach, we do not need to establish which phases of the rowing stroke correspond to a stable acceleration. We only assume that in synchronized rowers, these phases might be close. A second observation is that the collective mark contains a short-term memory concerning the overall closeness of the stroke accelerations of the rowers. Here, we can associate some semantics to the parameters of a mark. Small spatial and temporal decay may generate a Boolean processing: only almost identical rowing strokes can produce collective marking. Larger spatial and temporal decay allows distinguishing of different rowing strokes, up to a limit, which may cause growing collective marks with no stationary level.

**Figure 7.**
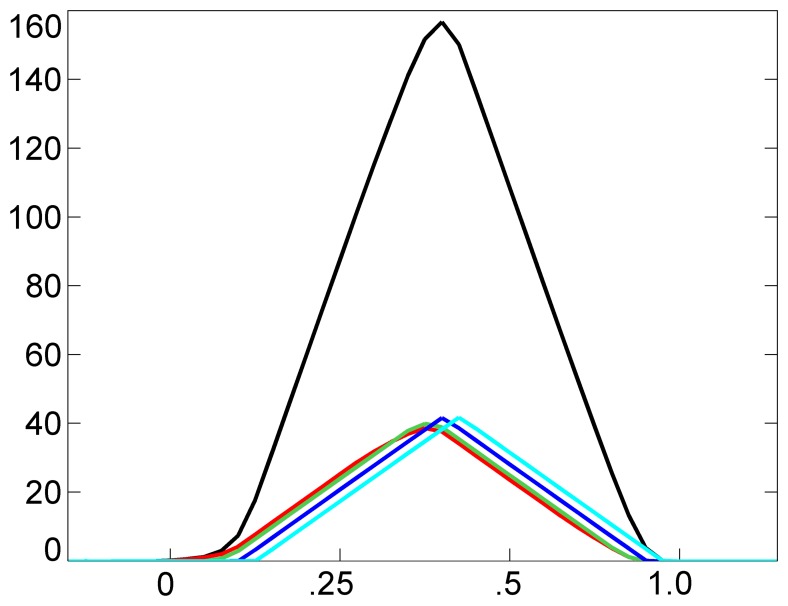
An example of close-to-optimal accumulated and collective marks, belonging to the pilot scenario, taken at *t* = 3.84 s, *I_MAX_* = 10, *ε* = 0.3 and *θ* = 0.75.

**Figure 8.**
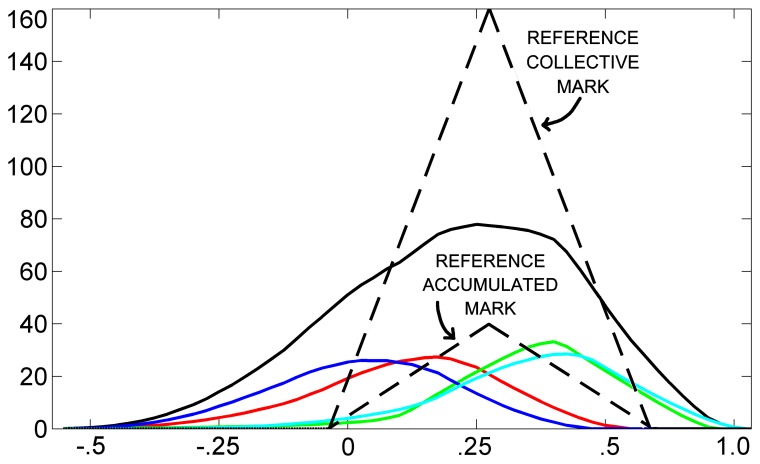
An example of marks produced by poorly synchronized rowing strokes (solid lines) with the reference marks (dashed lines).

Exploiting these observations, in the following, we discuss how a different type of agent can recognize the coordination of rowing strokes: the similarity agent. Basically, the similarity agent is responsible for assessing the similarity of accumulated and collective marks with respect to corresponding optimal marks. Let us first identify the optimal marks. Figure 7 shows an example of close-to-optimal marks, belonging to the pilot scenario (*N* = 4), taken at *t* = 3.84 s, *I_MAX_* = 10, *ε* = 0.3 and *θ* = 0.75. Indeed, from Formula (5), we can deduce that the optimal accumulated and collective marks are triangular marks with height equal to *I_MAX_*/(1 − *θ*) = 40 and *N* · *I_MAX_*/(1 − *θ*) = 160, respectively. Figure 8 shows an example of marks produced by poorly synchronized rowing strokes, which has been taken at *t* = 5.31s from the pilot scenario. Here, marks are not stationary, because their shape is very far from the triangular one. Here, the optimal (say, reference) accumulated and collective marks can be defined as triangular marks, with the centers placed at the barycenter of the current collective mark. In the figure, optimal accumulated and collective marks are also shown, with a dashed line.

**Figure 9.**
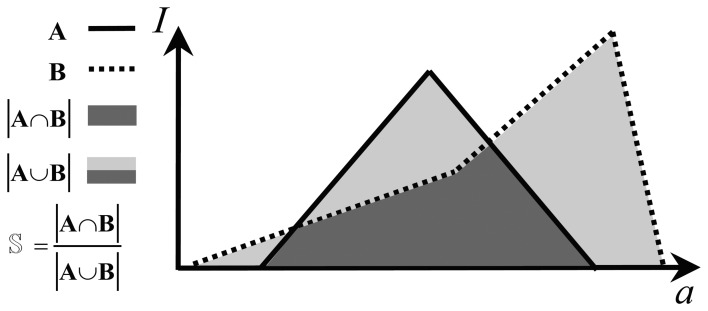
A visual representation of the similarity between two marks.

More formally, Figure 9 shows the similarity logic of the agent. Given a reference mark, *A*, and the current mark, *B*, their similarity is a real value calculated as the area covered by their intersection (colored dark gray in the figure) divided by the area covered by the union of them (colored light and dark gray). The lowest similarity is zero, *i.e.*, for marks with no intersection; the highest is one, *i.e.*, for identical marks. It is worth noting that accumulated and collective marks do not have, in general, a triangular shape. In conclusion, given *N* distinct accumulated marks, the similarity agent computes *N* individual similarities between each of the accumulated marks and the reference mark and a collective similarity between the collective mark and the reference collective mark.

**Figure 10.**
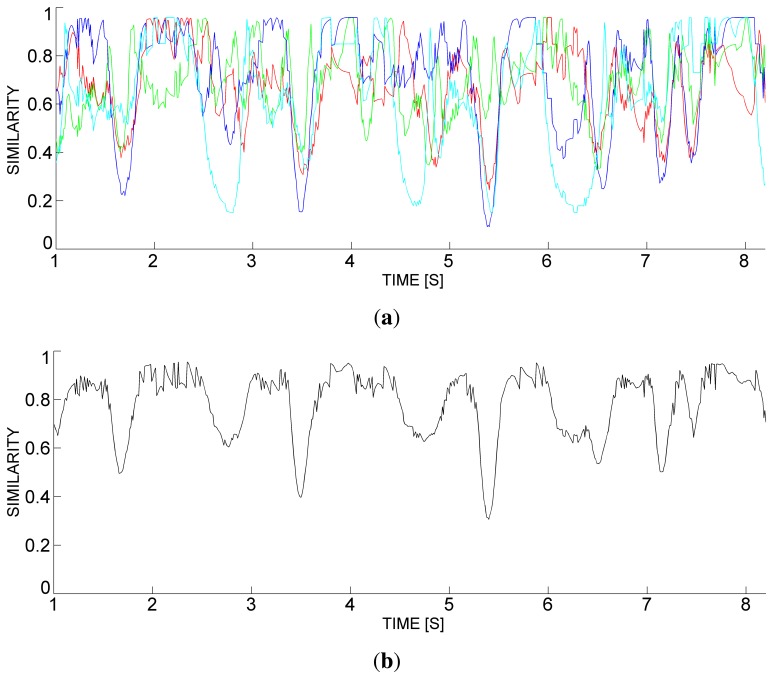
Similarity of the accumulated marks (**a**) and collective mark (**b**) with their references, in the pilot scenario

Table 1 shows the basic definitions that the agent uses for assessing the similarity of marks [30]. For instance, the similarity in the examples of Figures 7 and 8 is 0.950 and 0.556, respectively. As a result, Figure 10a and b show the similarity of accumulated and collective marks for the pilot scenario. Finally, the dissimilarity is calculated as the complement of similarity, according to Formula (vi) in Table 1.

### 4.4. The Granulation Processing Level

In this section, an attempt is made to establish some general transformation process that produces a human readable asynchrony starting from dissimilarity. In fact, the dissimilarity signal produced so far cannot be provided to the coach for an easy interpretation. In order to achieve accessibility, relevant information should be presented for actively supporting the decision process of the coach. More specifically, we focus on the concept of *information granulation* of a time series.

The process of information granulation is a vehicle of abstraction leading to the emergence of high-level concepts. More specifically, information can be granulated over predefined time intervals, giving rise to temporal granulation, but also, over the sensing variable (*i.e.*, acceleration), giving rise to sensing granulation. Information granules need to be *stable*, meaning that they have to retain their identity in spite of some small fluctuations occurring within the experimental data, as any judgment of an experienced coach. Further, information granules need to be *distinguishable*, meaning that their identities should be distinct enough from each other.

In user-oriented granulation, the user (*i.e.*, the coach) identifies the parameters of the information granules, according to his supervisory process. Proceeding with a given window of granulation, we propose some basic transformation. The interested reader may refer to [31] for a detailed study. More specifically, our objective is to construct crew and rower asynchrony descriptors that can be legitimized by the direct experience of a coach. The problem can be posed in the following way; given a collection of numeric dissimilarity data, let us say, $\text{D}=d_{j}^{\left(i\right)}\in {\mathbb{R}}^{(N+1)\times T}$, where $d_{0}^{\left(i\right)}$ and $d_{j}^{\left(i\right)}$ are the collective and the (*j*-th) individual dissimilarity at the *i*-th instant of time. A granulation process provides a collection of asynchrony data suitable for a specific *Performance Improvement Initiative* (PII) taken by the coach during a training process. Let us say: $\Gamma {\left(\text{D}\right)}_{\text{PII}}=\{\sigma_{j}^{\left(i\right)}\}\in {\mathbb{R}}^{(N+1)\times T}$, where $\sigma_{0}^{\left(i\right)}$ and $\sigma_{j}^{\left(i\right)}$ are the collective and the (*j*-th) individual asynchrony at the *i*-th instant. Hence, Γ(D) is a *performance indicator* with an intuitive interpretation based on the experimental evidence of an experienced coach. In brief, the PII represents a training process aimed at improving a performance indicator. Such a performance indicator is calculated via a granulation of the asynchrony measure.

In the following, we consider both local (online) and global (offline) PIIs, in order to monitor rowing performance within temporal windows of different scales. For this purpose, the process of granulation is established by determining the window of granulation and its numeric representative as a descriptor. A basic descriptor that can be considered is the simple moving average (SMA), *i.e.*, the unweighted mean of the previous Δ samples:

(7) $$\Gamma (\text{D})=\{\sigma_{j}^{\left(i\right)}\}=\{\begin{array}{cc} \text{undefined} & \text{if}\;i\leq \Delta \\ \frac{1}{\Delta }\sum_{k=0}^{\Delta -1}d_{j}^{\left(i-k\right)} & \text{if}\;i\geq \Delta \end{array}$$

where Δ is the temporal window. We use Formula (7) with two different scales of granulation, *i.e.*, a *macro-granulation* for a global (offline) PII and a *micro-granulation* for a local (online) PII.

**Figure 11.**
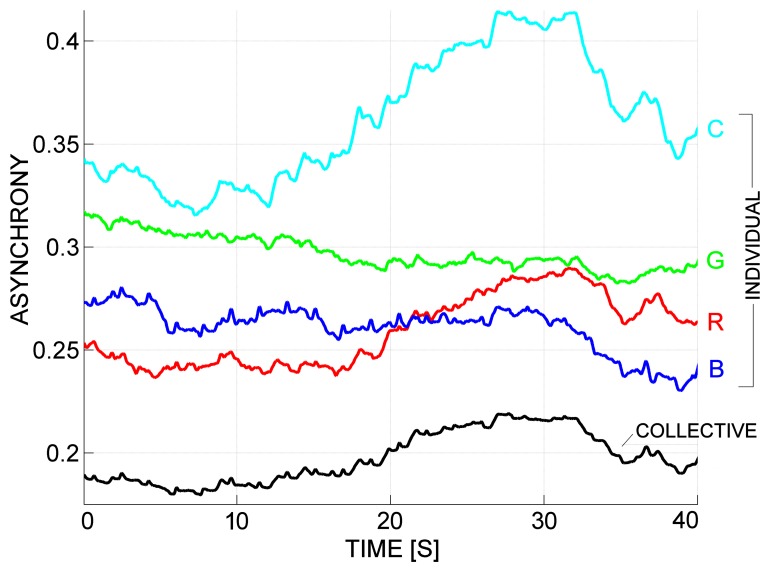
A scenario 40 s long, with individual (in color) and collective (in black) asynchrony, as a result of a macro-granulation with Δ = 2,000.

Figure 11 shows a scenario 40 s long, with individual (in color) and collective (in black) asynchrony, as a result of a macro-granulation process with Δ = 2,000. Here, we involved a crew of four rowers, named *Cyan* (C), *Red* (R), *Blue* (B) and *Green* (G). The following considerations can be easily made: (i) rowers C and G have the worst performance; (ii) rowers C and R sensibly diminished their performance in the second half of the scenario; and (iii) the collective performance sensibly diminished in the second half of the scenario.

**Figure 12.**
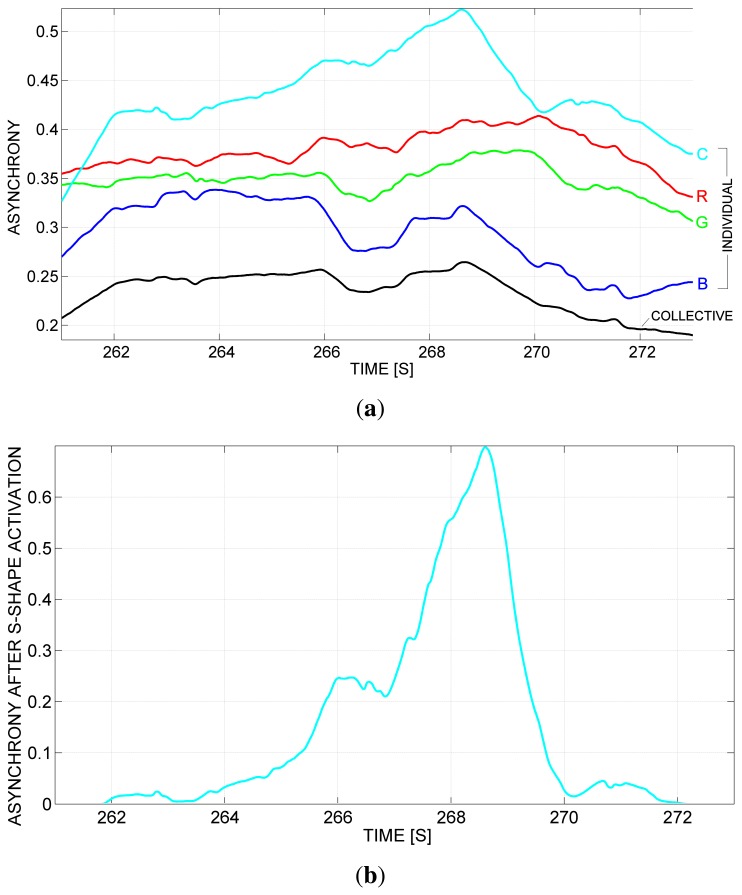
(**a**) A scenario of approximately 12 s, with individual (in color) and collective (in black) asynchrony, as a result of a micro-granulation with Δ = 800; (**b**) the same scenario after s-shape activation with *α* = 0.4 and *β* = 0.6.

**Figure 13.**
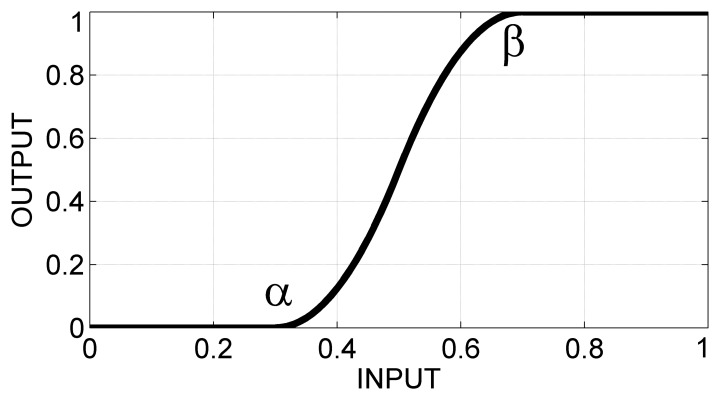
An *s*-shape activation function, with *α* = 0.3 and *β* = 0.7.

Figure 12a shows another scenario of asynchrony, resulting from a micro-granulation process with Δ = 800. In order to achieve a better distinction of the critical phenomena, a further sensing granulation has been performed, by applying an *s*-shaped activation function (Figure 13) with *α* = 0.4 and *β* = 0.6, considering the following definition:

(8) $$f(x;\alpha ,\beta )\{\begin{array}{ll} 0 & \text{if}\;x\leq \alpha \\ 2\cdot \frac{{\left(x-\alpha \right)}^{2}}{{\left(\beta -\alpha \right)}^{2}} & \text{if}\;\alpha \leq x\leq \frac{\alpha +\beta }{2}\\ 1-2\cdot \frac{{\left(x-\beta \right)}^{2}}{{\left(\beta -\alpha \right)}^{2}} & \text{if}\;\frac{\alpha +\beta }{2}\leq x\leq \beta \\ 1 & \text{if}\;x\geq \beta \end{array}$$

Figure 12b shows the resulting signal. As an effect of the sensing granulation, low values are further decreased, whereas higher values are further amplified, in order to evidence major discrepancies from the crew. Here, for example, the following considerations can be made: rower C increasingly loses their synchrony with respect to the group, starting from about the 264-*th* second and up to about the 269-*th* second, and, subsequently, regains synchrony.

In order to be provided to the aural display, the asynchrony signal has been also sonified [32]. In particular, our sonification scheme is aimed at producing a pure tone with a fixed amplitude and whose frequency is related to the asynchrony signal. More specifically, the frequency is calculated so as to produce musical notes of the diatonic scale. For this purpose, the input value is quantized on 12 values, ranging from 220 Hz to 440 Hz, corresponding to the musical notes *A3* and *A4*, respectively. The quantization function takes as input a continuous value, *σ*, between zero and one, and provides as output the quantized exponent, ⌊*σ* · 12⌋/12, where ⌊·⌋ stands for the truncation operator. More formally:

(9) $$\text{Sonification}(\sigma )=C_{0}\sin(2\pi \nu ),\;\nu =220\cdot 2^{\left\lfloor \sigma \cdot 12\right\rfloor /12}$$

where *C*_0_ is an amplitude constant and *σ* is the current asynchrony value. In order to perform a qualitative assessment of this sonification schema, an excerpt of sonification of the signal in Figure 12b can be publicly downloaded and listened to (http://tweb.ing.unipi.it/sonification.mp3).

## 5. The Deployment of the MARS System Architecture

The MARS system is not intended to substitute or replace the advice of the coach when conducting the training session. Instead, the system is aimed at helping the coach to better perceive the crew coordination. In our opinion, the fact that the MARS system performs as good as a trainer is indeed a primary goal and a good achievement.

**Figure 14.**
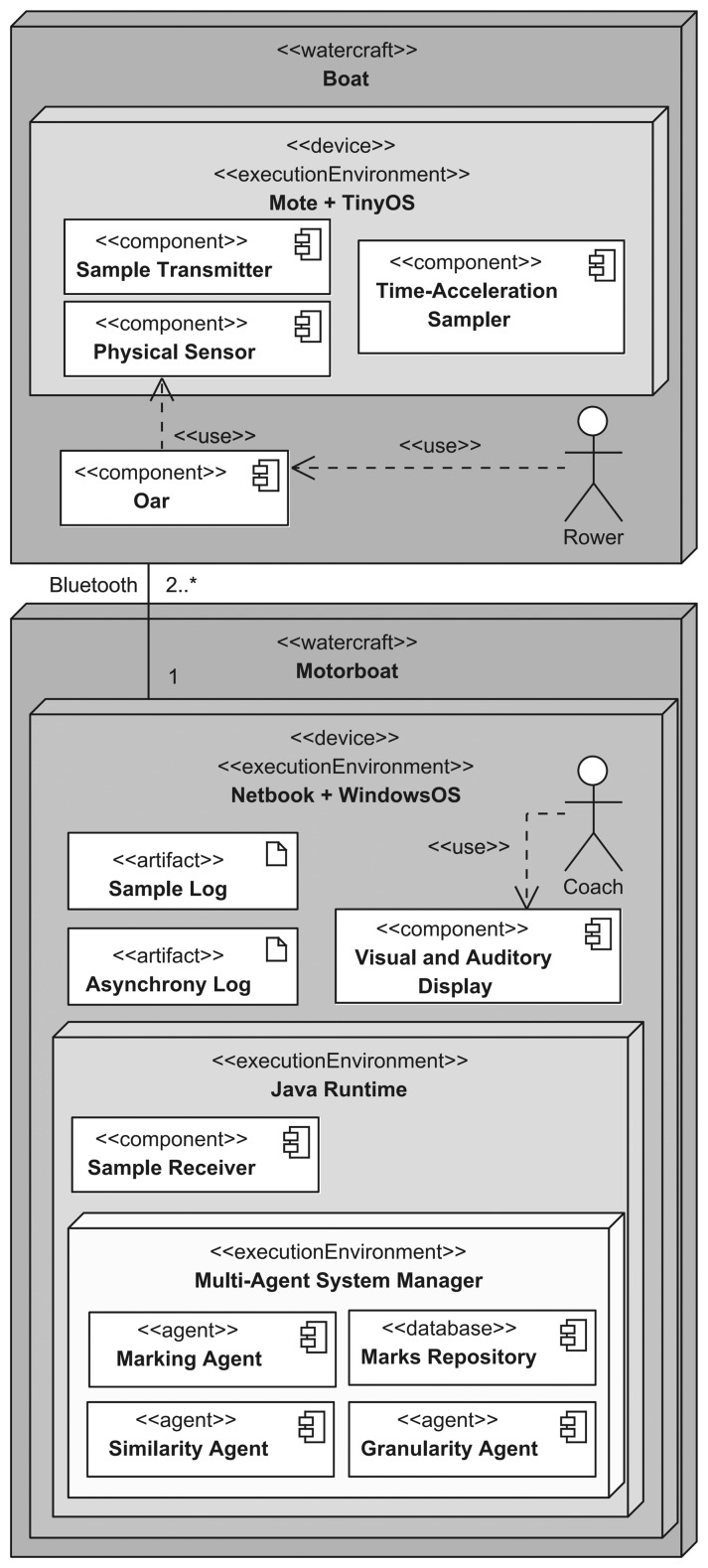
MARS, overall system architecture.

Figure 14 shows a UML (Unified Modeling Language) deployment diagram of the MARS system architecture. Here, there are two device categories, *i.e.*, *Mote* and *Netbook*, which reside on the boat and the motorboat, respectively There are many motes taking part in a sensing unit, each managed via the *TinyOS* operating system as an autonomous execution environment. There is a single netbook managed via the *Windows OS*, which hosts the tracking, processing and displaying units. On the boat, each *Rower* interacts indirectly with the *Physical Sensor* of a mote via his *Oar*. In the mote, the *Time-Acceleration Sampler* processes and records time and acceleration data from the physical sensor, whereas the *Sample Transmitter* component sends data to the Tracking Unit. On the motorboat, the coach is provided with asynchrony measures via the *Visual and Auditory Display* component, *i.e.*, the netbook display and the headphone, respectively. On the tracking unit, the *Sample Receiver* (a Java-based component) is provided with the data coming from motes. Such data are stored in the *Sample Log*. The processing unit is entirely based on the Java-based *Multi-Agent Systems Manager*, which hosts the various agents, and the *Marks Repository* implementing the mark properties. The Multi-Agent Systems Manager is based on Repast Simphony (http://repast.sourceforge.net), a Java-based modeling system supporting the development of interacting agents. It can be used as a GUI-based (user-driven) simulation environment, as well as an execution engine run from another Java application. As a final outcome, the processing unit provides the *Asynchrony Log*, which is the input for the *Visual and Auditory Display*. A single netbook can support up to a few tens of motes, via a wireless communication protocol based on Bluetooth. In particular, we used a total of four motes, one for each rower (as in the experiment, rowers used one oar each). In general, we can support a number of motes higher than the number of rowers.

## 6. Experimental Studies

The MARS research project got started by collaborating with a rowing team headed by a professional coach and comprising Olympic-level athletes. The current beta testing carried out with this team aims at assessing the effectiveness of the system in recognizing a reliable asynchrony measure, helping an experienced coach in his work. For this reason, a very expert coach has been chosen, so as to have reliable feedback. In this section, we report on experiments carried out to perform a check of the accuracy and repeatability of the MARS system. For such experiments, we involved a crew of four rowers: a novice, named *Cyan*(C), and three intermediate-level rowers, named *Red* (R), *Blue* (B) and *Green* (G). For each considered run, we made an acquisition session, involving the use of the MARS system and a parallel video recording of the session, respectively. In all sessions, the sampling frequency has been set to 100 Hz. Table 2 shows the main features of the considered runs. Different types of runs have been considered, in order to test the system on a variety of conditions. Each session is divided into observation slices. An observation slice is a temporal window of some seconds in which the coach expresses a level of asynchrony by observing rowers with the naked eye, namely, watching the video (possibly in slow motion) without using the MARS system. There are three possible levels of asynchrony that can be expressed: *Low* (L), *Medium* (M) and *High* (H).

For each session, the local (online) asynchrony has been processed, by using Δ = 2,000. Training and testing data are separated in the validation. More specifically, session *A* has been used as the tuning (training) session, whereas the other sessions are used for testing. Hence, during the first session, the parameters for the activation function have been set, according to the PII established by the coach, who was focused on the novice rower, Cyan. Once the coach finished his assessment of run *A*, the activation function parameters have been set in order to produce the corresponding outcome via the MARS system. In this process, *α* and *β* were set to 0.3 and 0.5, respectively. The setting of the two parameters is simple: starting from standard values, which can be easily adjusted with a very few trials, so as to pursue the reference values provided by an experienced coach. Table 3 shows the comparison between the coach and the system opinions. The values expressed in a column represent the extent to which the stroke of a single rower differs from the collective (emergent) behavior of the overall crew. The coach asynchrony values represent the human opinion provided by watching the athletes when rowing, ranging from L (Low) to H (High), whereas the system asynchrony values represent the system output computed by the multi-agent system, ranging from zero to one. The coach was asked to perform the analysis using a video playback. The numerical results provided by the MARS system can be located into corresponding classes used by the coach, using the following mapping: [0, 0.2] → *L*, (0.2, 0.5] → *M* and (0.5, 1.0] → *H*. As a result of the tuning session, the asynchrony produced by the system has become totally compliant with the coach classification.

Tables 4, 5, 6 and 7 show the results for the other sessions. By using the same parameters established in the tuning session, it can be noticed that in the testing sessions, the asynchrony values produced by the system are totally compliant with the corresponding coach opinions, thus confirming the effectiveness of the system.

First, it should be noted that the variability of asynchrony is almost entirely expressed by the novice (Cyan) rower, according to the PII of the coach. More specifically, in session *A*, the Cyan (C) rower loses his synchrony at the start of the observation slice (first five seconds) and close to its end, with a peak between the 40*^th^* and the 50*^th^* second. In session *B*, at the 31*^th^* second, both the Green and Cyan rowers lose their synchrony. It is worth nothing that the coach is not able to measure the difference between the two rowers, whereas the MARS system is able to assess that the Cyan performance is worse (0.40) with respect to the Green rower (0.22). In addition, at the 50*^th^* second, the Cyan rower loses his synchrony again. In session *C*, the Cyan rower is the unique rower who appreciably loses his synchrony with three peaks, between the 5*^th^* and the 10*^th^* second, between the 20*^th^* and the 25*^th^* second and between the 40*^th^* and the 45*^th^* second. A similar behavior can be observed in session *D*, where a relevant asynchrony is detected in the first five seconds. Finally, in session *E* is the Red rower, who, together with the Cyan rower, shows some performance drop between the 30*^th^* and the 40*^th^* second. Again, note how the system is able to provide a more precise measurement of the performance variability, which is, in any case, in agreement with the assessment given by the coach.

## 7. Conclusions and Future Work

In this paper, we presented MARS, a multi-agent system for assessing rowers' coordination via motion-based stigmergy. The system is able to provide online available feedback to the trainer. In the system, a sensing unit allows local sensing of the strokes via motes, a tracking unit collects and integrates the sensed data, a processing unit computes crew and athlete asynchrony and a displaying unit provides visual and aural asynchrony feedback to the coach. The processing unit is based on the emergent approach, in which software agents employ a stigmergic computing scheme to measure the extent of similarity between the behavior of the rowers. This paper shows both architectural and functional views. The MARS system was tested on real-world rowing scenarios, involving four athletes with different experience on a number of runs. The results obtained in terms of asynchrony demonstrate that the proposed scheme can be successfully applied in the field.

In this study, the MARS system was experimented upon by a rowing team headed by a professional coach and comprising Olympic-level athletes. Nevertheless, to ensure high-quality design, the system should be cross-validated against the coach's subjectivity. An important future development will be the recruitment of other experienced coaches and rowing teams for enabling a robust expert-driven validation. Moreover, as future work, we aim at designing a self-tuning module for the parameters that need a manual setting. We will also experiment with new sonification methods based on *earcons*, musical or vocal motifs/sounds that humans use to improve the aural display of the system.

## Figures and Tables

**Table 1.** Basic definitions for assessing the similarity of marks.

	**Definition**	**Name and properties**
(i)	$\left|I(a)\right|=\int_{-\infty }^{\infty }I(a)da$	Cardinality of a mark, *I*(*a*): a real number.
(ii)	$a*=\frac{\int_{-\infty }^{\infty }I(a)\cdot a\;da}{\int_{-\infty }^{\infty }I(a)da}$	Barycenter of a mark, *I*(*a*): a real number.
(iii)	*I_A_*(*a*) ∩ *I_B_*(*a*) = min (*I_A_*(*a*),*I_B_*(*a*))	Intersection of two marks, *I_A_*(*a*) and *I_B_*(*a*): a mark.
(iv)	*I_A_*(*a*) ∪ *I_B_*(*a*) = max (*I_A_*(*a*),*I_B_*(*a*))	Union of two marks, *I_A_*(*a*) and *I_B_*(*a*): a mark.
(v)	$S\;[I_{A}(a),I_{B}(a)]=\frac{\left|I_{A}(a)\cap I_{B}(a)\right|}{\left|I_{A}(a)\cup I_{B}(a)\right|}$	Similarity of two marks, *I_A_*(*a*) and *I_B_*(*a*): a real number.
(vi)	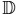 [*I_A_* (*a*), *I_B_* (*a*)] = 1 − 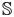 [*I_a_*(*a*),*I_B_*(*a*)]	Dissimilarity, complement of similarity: a real number.

**Table 2.** Runs and their main features.

**Run (session)**	**Total Samples**	**Average Strokes per Minute (SPM)**	**Number of Observation slices**	**Type of run**
*A*	6,500	33.4	13	inner-club competition
*B*	8,000	15.4	8	training
*C*	4,500	32.0	9	inner-club competition
*D*	5,000	31.2	10	inner-club competition
*E*	8,000	15.0	8	recovery

**Table 3.** Session *A*, coach and system assessment.

**Time (s)**	**Coach**				**MARS System**			
	
	R	G	B	C	R	G	B	C
5	L	L	L	M	0.02	0.00	0.00	0.23
10	L	L	L	L	0.00	0.01	0.00	0.14
15	L	L	L	L	0.00	0.01	0.00	0.19
20	L	L	L	L	0.00	0.00	0.00	0.04
25	L	L	L	L	0.00	0.02	0.00	0.02
30	L	L	L	L	0.00	0.00	0.00	0.01
35	L	L	L	L	0.00	0.00	0.00	0.09
40	L	L	L	M	0.00	0.00	0.00	0.32
45	L	L	L	H	0.00	0.00	0.00	0.70
50	L	L	L	H	0.02	0.00	0.00	0.76
55	L	L	L	M	0.00	0.00	0.00	0.44
60	L	L	L	L	0.00	0.00	0.00	0.01
65	L	L	L	L	0.00	0.00	0.00	0.00

**Table 4.** Session *B*, coach and system assessment.

**Time (s)**	**Coach**				**MARS System**			
	
	**R**	**G**	**B**	**C**	**R**	**G**	**B**	**C**
10	L	L	L	L	0.00	0.02	0.00	0.05
20	L	L	L	L	0.00	0.04	0.00	0.13
30	L	M	L	M	0.02	0.22	0.00	0.40
40	L	L	L	L	0.00	0.03	0.00	0.14
50	L	L	L	H	0.01	0.09	0.00	0.62
60	L	L	L	H	0.00	0.07	0.01	0.62
70	L	L	L	M	0.00	0.06	0.00	0.46
80	L	L	L	M	0.02	0.04	0.00	0.48

**Table 5.** Session *C*, coach and system assessment.

**Time (s)**	**Coach**				**MARS System**			
	
	**R**	**G**	**B**	**C**	**R**	**G**	**B**	**C**
5	L	L	L	M	0.09	0.02	0.00	0.28
10	L	L	L	H	0.03	0.01	0.00	0.55
15	L	L	L	M	0.00	0.03	0.00	0.33
20	L	L	L	M	0.00	0.03	0.00	0.50
25	L	L	L	H	0.00	0.09	0.03	0.89
30	L	L	L	L	0.01	0.01	0.00	0.14
35	L	L	L	L	0.02	0.00	0.00	0.01
40	L	L	L	L	0.01	0.00	0.00	0.11
45	L	L	L	H	0.00	0.05	0.00	0.51

**Table 6.** Session *D*, coach and system assessment.

**Time (s)**	**Coach**				**MARS System**			
	
	**R**	**G**	**B**	**C**	**R**	**G**	**B**	**C**
5	L	L	L	H	0.00	0.05	0.00	0.53
10	L	L	L	L	0.03	0.03	0.00	0.10
15	L	L	L	L	0.02	0.03	0.00	0.14
20	L	L	L	M	0.00	0.00	0.00	0.37
25	L	L	L	L	0.00	0.00	0.00	0.15
30	L	L	L	L	0.00	0.00	0.00	0.13
35	L	L	L	M	0.00	0.01	0.00	0.20
40	L	L	L	L	0.00	0.01	0.00	0.10
45	L	L	L	L	0.00	0.00	0.00	0.01
50	L	L	L	L	0.00	0.00	0.00	0.00

**Table 7.** Session *E*, coach and system assessment.

**Time (s)**	**Coach**				**MARS System**			
	
	**R**	**G**	**B**	**C**	**R**	**G**	**B**	**C**
10	L	L	L	L	0.00	0.00	0.00	0.00
20	L	L	L	M	0.00	0.01	0.00	0.23
30	L	L	L	L	0.05	0.07	0.00	0.00
40	M	L	L	H	0.32	0.14	0.02	0.71
50	L	L	L	M	0.10	0.03	0.00	0.26
60	L	L	L	L	0.00	0.06	0.00	0.02
70	L	L	L	L	0.00	0.01	0.00	0.00
80	L	L	L	L	0.00	0.11	0.00	0.12
